# Cellular membranes of the related fission yeast species differ in their dipole potential

**DOI:** 10.1016/j.bpr.2026.100283

**Published:** 2026-08-26

**Authors:** Bhagyashree Dasari Rao, Snezhana Oliferenko

**Affiliations:** 1 Randall Centre for Cell and Molecular Biophysics, School of Basic and Medical Biosciences, King’s College London, London, SE1 1UL, UK; 2 The Francis Crick Institute, 1 Midland Road, London, NW1 1AT, UK

## Abstract

The dielectric environment and membrane dipole potential are key electrostatic properties of biological membranes, which modulate the function of membrane-associated proteins. If and how these properties are shaped by species-specific cellular lipid landscapes is a fundamental question. Here we use two related fission yeast species, *S. pombe* and *S. japonicus*, which exhibit remarkable differences in membrane lipid composition, to address this problem. *S. pombe* synthesizes membranes from largely unsaturated glycerophospholipids with 18- and 16-carbon long fatty acyl chains and the major fungal sterol ergosterol. Its relative *S. japonicus* produces abundant saturated asymmetrical glycerophospholipids that contain a medium chain fatty acyl C10:0 at the *sn-2* position of the glycerol backbone. Alongside ergosterol, its membranes contain the sterol mimic diplopterol, produced by a horizontally transferred squalene hopene cyclase of a bacterial origin. Using fluorescence lifetime imaging of the solvatochromic dye di-4-ANEPPDHQ we show that *S. pombe* and *S. japonicus* exhibit comparable dielectric environment. Interestingly, dipole potential measurements using the voltage-sensitive probe di-8-ANEPPS show that *S. japonicus* membranes have higher dipole potential relative to *S. pombe*. The in vivo measurements of sterol- and diplopterol-lacking *S. japonicus* mutants supported by experiments with model membranes and *S. pombe* retroengineered to produce the C10-containing glycerophospholipids, indicate that both ergosterol and the saturated asymmetrical glycerophospholipids support high membrane dipole potential. Our results suggest that membrane physicochemical properties result from a combination of lipid composition, packing and interfacial electrostatics and point to possible avenues in exploring the evolutionary differences in membrane protein function.

## Introduction

Lipids are essential building blocks of cells. They form biological membranes delimiting cells and subcellular compartments, modulate functions of membrane-associated proteins, participate in cellular signalling, and facilitate energy production^1–4^. Eukaryotic lipids exhibit large structural diversity, from several hundred species in model yeasts to up to thousands in mammals^5–7^. Glycerophospholipids (GPLs) that form the structural scaffold of cellular membranes differ in their head groups and the length and degree of unsaturation of the fatty acyl (FA) chains. Depending on their chemical structure, glycerophospholipids endow membranes with different physicochemical properties; as an example, glycerophospholipid unsaturation decreases membrane order^8,9^. Triterpenoid lipids sterols and sphingolipids interact with glycerophospholipids and membrane-associated proteins, further modifying membrane properties^10,11^. Lipid composition varies between species, cell types, intracellular organelles and even within individual membranes. How specific lipids contribute to membrane physicochemical properties and cellular physiology remains an important question.

The widely used model fission yeast *Schizosaccharomyces pombe* (*S. pombe*) and its relative, the emerging model system *Schizosaccharomyces japonicus* (*S. japonicus*), exhibit fascinating differences in membrane lipid composition. *S. pombe* synthesizes largely di-unsaturated and mono-unsaturated glycerophospholipid species, where most FA chains are 18 and 16 carbon atoms long. On the other hand, *S. japonicus* produces a substantial amount of saturated asymmetrical glycerophospholipids, usually with the long C18:0 FA at the *sn-1* position and the medium C10:0 FA at the *sn-2* position of the glycerol backbone^12^. Furthermore, *S. japonicus* produces abundant hydroxyl-containing triterpenoid diplopterol, which belongs to the class of hopanoids, the bacterial mimics of eukaryotic sterols. It also synthesizes minor hopanoids, including the non-polar diploptene^13,14^. Hopanoids in *S. japonicus* are produced by a horizontally transferred squalene hopene cyclase (SHC) of a bacterial origin^13,14^. Unlike sterols, the synthesis of which consumes large amounts of molecular oxygen^15^, SHC enzymes do not require oxygen for hopanoid production^16^. *S. japonicus* produces diplopterol already in normoxia, alongside the major fungal sterol ergosterol, and switches completely to the production of hopanoids in anoxia, when sterol synthesis is not possible. On the other hand, *S. pombe*, like most eukaryotes, relies exclusively on sterol (ergosterol) production^12,14^. The acquisition of SHC has allowed *S. japonicus* to thrive in the absence of oxygen^13,14,17,18^, unlike *S. pombe*, which is an obligate aerobe. Interestingly, beyond anaerobic growth, hopanoids appear to contribute to growth at higher temperatures both in *S. japonicus* and upon their introduction to *S. pombe*^14^, suggesting that they may broadly modify membrane physiology.

Our recent work identified the glycerophospholipid fatty acyl asymmetry, prevalent in *S. japonicus*, as a critical feature allowing to accommodate both ergosterol and diplopterol in membranes, at least in vitro^14^. Cryo-EM measurements revealed that the asymmetrical saturated C18:0-C10:0 (1-stearoyl-2-decanoyl-sn-glycero-3-phosphocholine, SDPC) formed thinner membranes in comparison to symmetrical unsaturated C16:0-C18:1 (1-palmitoyl-2-oleoyl-sn-glycero-3-phosphocholine, POPC). Two-component mixtures of SDPC or POPC with either triterpenoid were relatively disordered as compared to DPPC/ergosterol gel-phase membranes, but ergosterol supported higher membrane order relative to diplopterol. In ternary lipid mixtures with DPPC, SDPC supported phase separation in the presence of either ergosterol or diplopterol, whereas only ergosterol could induce phase separation in the presence of POPC^14^. Interestingly, the measurements of membrane order in live cells using C-laurdan indicated that – as compared to *S. pombe* – the nuclear membrane and the plasma membrane appeared more ordered in *S. japonicus*, with both diplopterol and ergosterol contributing to maintaining membrane order^14^.

Beyond membrane order, dielectric properties of the membrane environment and dipole potential play key roles in membrane organization and the function of membrane-associated proteins^19^. Here, we investigate these parameters in *S. japonicus* and *S. pombe* using the voltage-sensitive probes, di-4-ANEPPDHQ and di-8-ANEPPS^20,21^. Using confocal microscopy-based imaging of live cells and spectroscopic measurements of model membranes we determine the contribution of diplopterol and ergosterol, as well as the glycerophospholipid architecture to these membrane properties. Our results indicate that the dielectric environment and dipole potential exhibit a non-linear relationship and emerge from a combination of lipid packing, composition and interfacial electrostatics.

## Materials and Methods

### Fission yeast strains and growth conditions

The prototrophic *S. japonicus* and *S. pombe* strains used in this work are listed in Supplemental Table S1. Cells were grown in the modified minimal yeast nitrogen base (YNB) medium (YNB containing 111 mM glucose, 14.7mM potassium hydrogen phthalate, 15.5mM disodium hydrogen phosphate, and the following supplements: adenine (93.75 mg/l), uracil (75 mg/l), histidine (75 mg/l) and leucine (75 mg/l), as previously^14^.

### Labelling fission yeast cells with voltage sensitive dyes

Cells were routinely pre-cultured overnight, diluted to OD_595_ 0.1 and grown in minimal modified YNB medium in 200 rpm shaking incubators at 30°C, to reach mid-exponential phase (OD_595_ 0.4-0.6).

1ml cell culture was concentrated to 30 μl by centrifugation at 1500 x g for 1 min. Cells were labelled with either 2 μM di-8-ANEPPS for dipole potential measurements or 2 μM di-4-ANEPPDHQ for FLIM measurements, and incubated at 25°C for 30 min. Following incubation, cells were centrifuged at 1500 x g for 1 min, resuspended in fresh modified YNB medium and mounted on a glass slide. Acquisitions were performed immediately after sample preparation.

### Fluorescence Lifetime Imaging measurements in live fission yeast cells

Confocal fluorescence lifetime imaging (FLIM) measurements was performed on an inverted Stellaris 8 Falcon (Leica) microscope (Microscopy Innovation Centre, King’s College London) equipped with the white-light laser (440−790 nm) using a 63x/1.4 NA oil immersion objective under 1 airy condition. Di-4-ANEPPDHQ was excited at 468 nm (laser intensity: 0.21%) and was detected in the range of 500-830 nm (Hyd X3 detector), with a pixel dwell time of 5.6 μs. Images were acquired with a resolution of 256 × 256 pixels and frame repetitions was set to 50. Raw data were analysed using the dedicated LAS X FLIM analysis software. Regions of interest (ROIs) in the plasma or nuclear membrane were fit to a single exponential decay model using the n-exponential tail fit, and fluorescence lifetimes <τ> were computed by the software, according to the equation below:

$$I(t)=Ae^{-t/\tau }+C$$

where *I(t)* represents the intensity at time *t, A* is the fluorescence decay amplitude at *t* = 0, τ is the fluorescence lifetime (the time at which amplitude *A* decays to *A/e*, ~37%), and *C* is the offset value. Fit quality was assessed using the χ^2^ values generated by LAS X. Representative fluorescence decay profiles, together with the corresponding fluorescence lifetime (τ) and χ^2^ values obtained from the LAS X fits, are provided in Supplemental Fig. S1.

### Live confocal microscopy-based measurements of dipole potential

Ratiometric imaging was carried out on a Nikon AXR inverted confocal microscope with NSPARC1 enabled with 2 GaAsP detectors with a 63×/1.4 NA oil immersion objective under 1 airy condition. Labelled cells were imaged using two excitation wavelengths (445 and 514 nm) with a 650-717 nm emission band pass in both cases. Keeping the emission wavelength at the red edge of the fluorescence spectrum was previously shown to rule out membrane fluidity effects^22^. All measurements were done at room temperature (~25°C). The fluorescence intensity ratio (R), defined as the ratio of fluorescence intensities at an excitation wavelength of 445 nm to that at 514 nm was calculated using Fiji/ImageJ, while the.nd2 format images were analyzed using a custom macro^23^. For quantitative analysis, ROIs were defined on the raw fluorescence images rather than on the pseudocolour ratio maps. Plasma-membrane ROIs were placed at cell tips and NE/ER ROIs were drawn over clearly resolved segments of the nuclear membrane. Identical ROI coordinates were applied to both fluorescence channels. Background fluorescence was determined and subtracted separately for each channel, and the fluorescence ratio (R) was calculated from the mean background-corrected fluorescence intensities within each ROI. Poorly resolved regions and regions with insufficient fluorescence signal were excluded. Pseudocolour ratiometric maps were generated for qualitative visualization only and were not used for quantitative measurements.

### Preparation of large unilamellar vesicles

Large unilamellar vesicles (LUVs) were prepared as described previously with slight modifications^24^. Specifically, for dipole potential measurements, 300 nmol lipids and 3 nmol di-8-ANEPPS (lipid-to-probe ratio 300:1) were mixed and dried using a nitrogen stream while being warmed gently at 37°C. Lipid samples were dried in vacuum for 3 h, followed by hydration at 60°C for 1 h in 1ml of buffer A (10mM sodium phosphate, 150mM NaCl, pH 7.4) for dipole potential measurements. Samples were vortexed for 1 min to form homogeneous multilamellar vesicles (MLVs). LUVs were prepared by extrusion using Avestin Liposofast Extruder (Ottawa, Canada) as previously described^25^. Briefly, MLVs were freeze-thawed five times using liquid nitrogen and extruded through polycarbonate filters (pore diameter of 100 nm) mounted on extruder fitted with Hamilton syringes (Hamilton Company, Reno, NV). The samples were subjected to 21 passes for all liposomes on a 55°C hot plate. LUV sizes were measured using dynamic light scattering (Malvern Zetasizer Nano ZS). Samples were kept overnight at 25°C before measurements.

### Dipole potential measurements in liposomes

Spectroscopic dipole potential measurements were performed at 25°C on a FP-8300 spectrofluorometer (Jasco, West Yorkshire, UK) using a quartz cuvette with 1-cm path length. Fluorescence excitation spectra of the voltage sensitive probe di-8-ANEPPS was measured from 400 to 600 nm, while keeping the emission wavelength fixed at 670 nm. Fluorescence emission spectra of the probe were measured from 490 to 700 nm, while keeping the excitation at 470 nm. All experiments were carried out using excitation and emission slits with a bandpass of 5 nm. The fluorescence intensity ratio (R), defined as the ratio of fluorescence intensities at an excitation wavelength of 420 nm to that at 510 nm provides an estimate of the membrane dipole potential. R is related to the dipole potential (Ψ_d_) according to the linear relationship below^26–28^:

$$\psi_{\text{d}}=(\text{R}+0.3)/(4.3\times 10^{-3})$$

### Statistics and Reproducibility

The statistical details of experiments, including the number of biological replicates can be found in Figure Legends and Supplemental Information. Data were analysed using unpaired *t*-test statistical analysis, unless mentioned otherwise. All plots were generated using GraphPad Prism 11.

## Results

### *S. pombe* and *S. japonicus* membranes exhibit comparable dielectric environment

We first set out to probe the dielectric environment in the membranes of live *S. pombe* and *S. japonicus* cells by fluorescence lifetime imaging (FLIM) of the solvatochromic dye di-4-ANEPPDHQ. Fluorescence lifetime is determined by the excited-state relaxation dynamics of the probe and depends on its immediate molecular environment^29,30^. Although di-4-ANEPPDHQ is sometimes used as a probe for membrane order, its spectral emission characteristics and solvatochromic behaviour correlate with dielectric properties of the local membrane environment^31^. We measured the di-4-ANEPPDHQ fluorescence lifetimes at the plasma membrane (PM) and the endoplasmic reticulum (ER). Since in fission yeasts, the peripheral ER is tightly associated with the plasma membrane at the lateral cell cortex^32–34^, plasma membrane measurements were performed at cell tips, where the ER tends to be spatially separated from the plasma membrane. Accordingly, the nuclear envelope (NE) was used as proxy for the ER.

**Figure 1:**
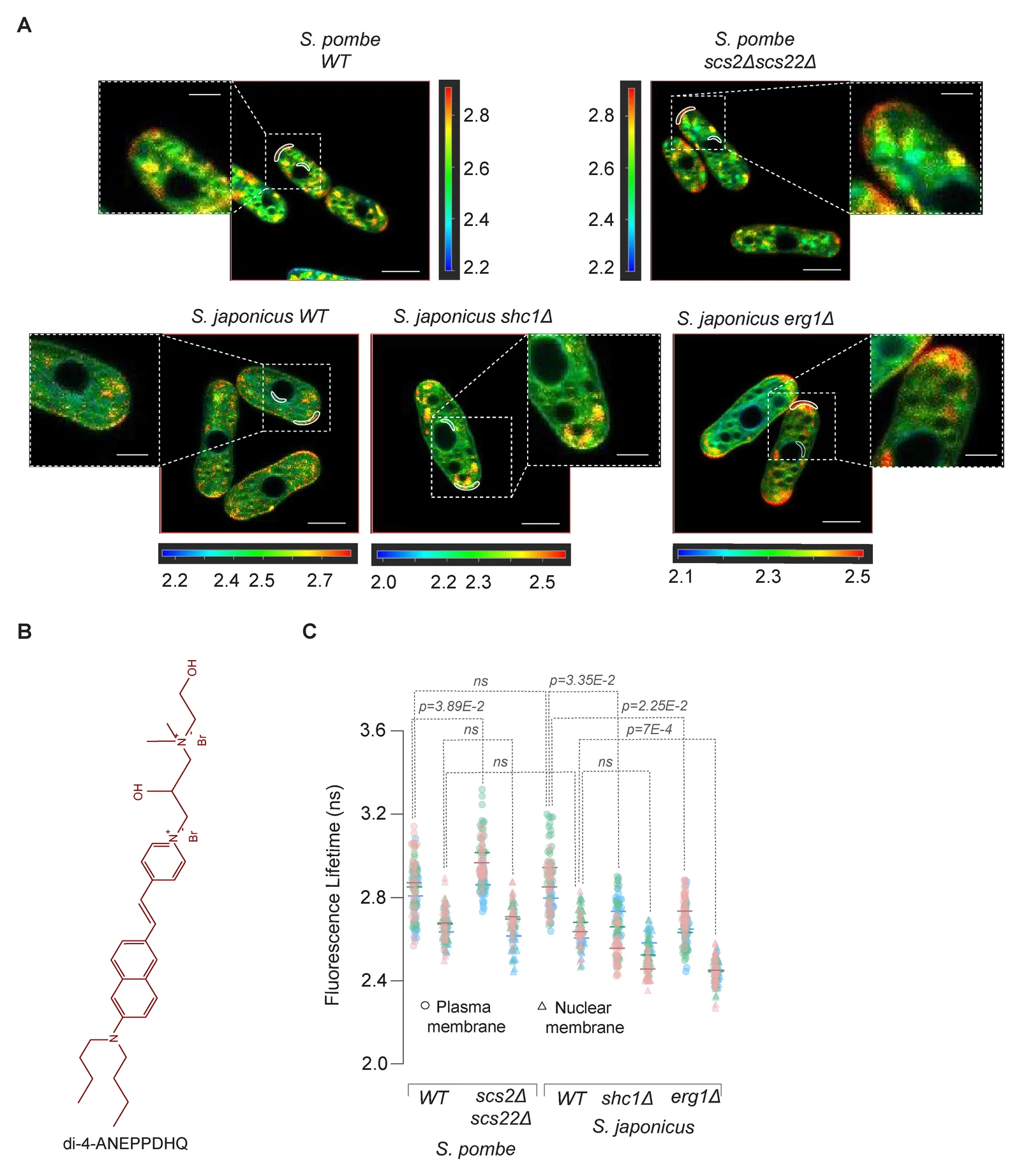
Di-4-ANEPPDHQ FLIM measurements reveal comparable dielectric environment in *S. pombe* and *S. japonicus* membranes. (**A**) Representative single plane pseudo-coloured FLIM images of wild-type (*WT*) and *scs2Δscs22Δ* mutant *S. pombe* strains (top panel). Representative FAST FLIM images of *WT S. japonicus* and mutants deficient in synthesizing either ergosterol (*erg1Δ*) or diplopterol (*shc1Δ*) (bottom panel). The corresponding color-coded scale bars for fluorescence lifetime (selected automatically for best contrast) are shown, where blue shows shorter fluorescence lifetime and red shows longer fluorescence lifetime. Images were obtained after processing raw data in the LAS X FLIM analysis software. Dashed boxes indicate regions shown at higher magnification. Representative regions of interest (ROIs) corresponding to the plasma membrane at the cell tip and the nuclear membrane are indicated. Scale bar represents 5 µm in the main images and 2 µm in the magnified images. (**B**) Chemical structure of the solvatochromic dye di-4-ANEPPDHQ used for FLIM measurements. (**C**) A plot representing individual fluorescence lifetimes at the plasma membrane and at the NE/ER in cells of specified genotypes. The fluorescence lifetime values were obtained from a single exponential fitting of the decay curves acquired from the indicated representative regions of interest (ROIs). Data points represent measurements from individual cells, and data from three independent biological replicates are colour coded. Horizontal lines indicate the mean of each biological replicate. *p* values were obtained by two-tailed unpaired parametric *t*-tests, using the means of the three independent biological replicates (n = 3).

Our measurements showed comparable fluorescence lifetimes for di-4-ANEPPDHQ in membranes of the two species (Fig. 1A-C and Supplemental Fig. S1). As expected, the values for the plasma membrane were consistently higher as compared to the nuclear envelope / endoplasmic reticulum.

We validated the FLIM approach using *S. pombe* cells lacking the two VAMP-associated proteins (VAP) Scs2 and Scs22, which promote the anchoring of the ER to the plasma membrane and play a role in phosphoinositide (PI) homeostasis^35,36^. The loss of the two VAPs leads to an increase in the levels of phosphatidylinositol-4-phosphate (PI4P) at the plasma membrane^36^. PI4P is an anionic lipid that powers strong electrostatic interactions, controlling interaction and function of membrane-associated proteins, and potentially contributing to membrane potential^37–40^. We indeed observed longer fluorescence lifetime for di-4-ANEPPDHQ specifically at the plasma membrane of *scs2Δscs22Δ S. pombe* cells relative to the wild type (Fig. 1A, C).

In *S. japonicus*, the lack of either hopanoids (due to the deletion of *shc1*, the gene encoding the SHC) or sterols (due to the deletion of *erg1* encoding the squalene monooxygenase Erg1) led to a comparable decrease in di-4-ANEPPDHQ fluorescence lifetime at the plasma membrane (Fig. 1A, C). Although the fluorescence lifetime values at the NE tended to be lower than in the wild type in both mutants, the difference reached statistical significance only in the absence of ergosterol (Fig. 1A, C). These results are broadly consistent with our previous C-laurdan measurements showing reduced membrane order upon depletion of either triterpenoid, although the extent of perturbation differs at the nuclear membrane^14^.

### Dipole potential differs in *S. pombe* and *S. japonicus* membranes

**Figure 2:**
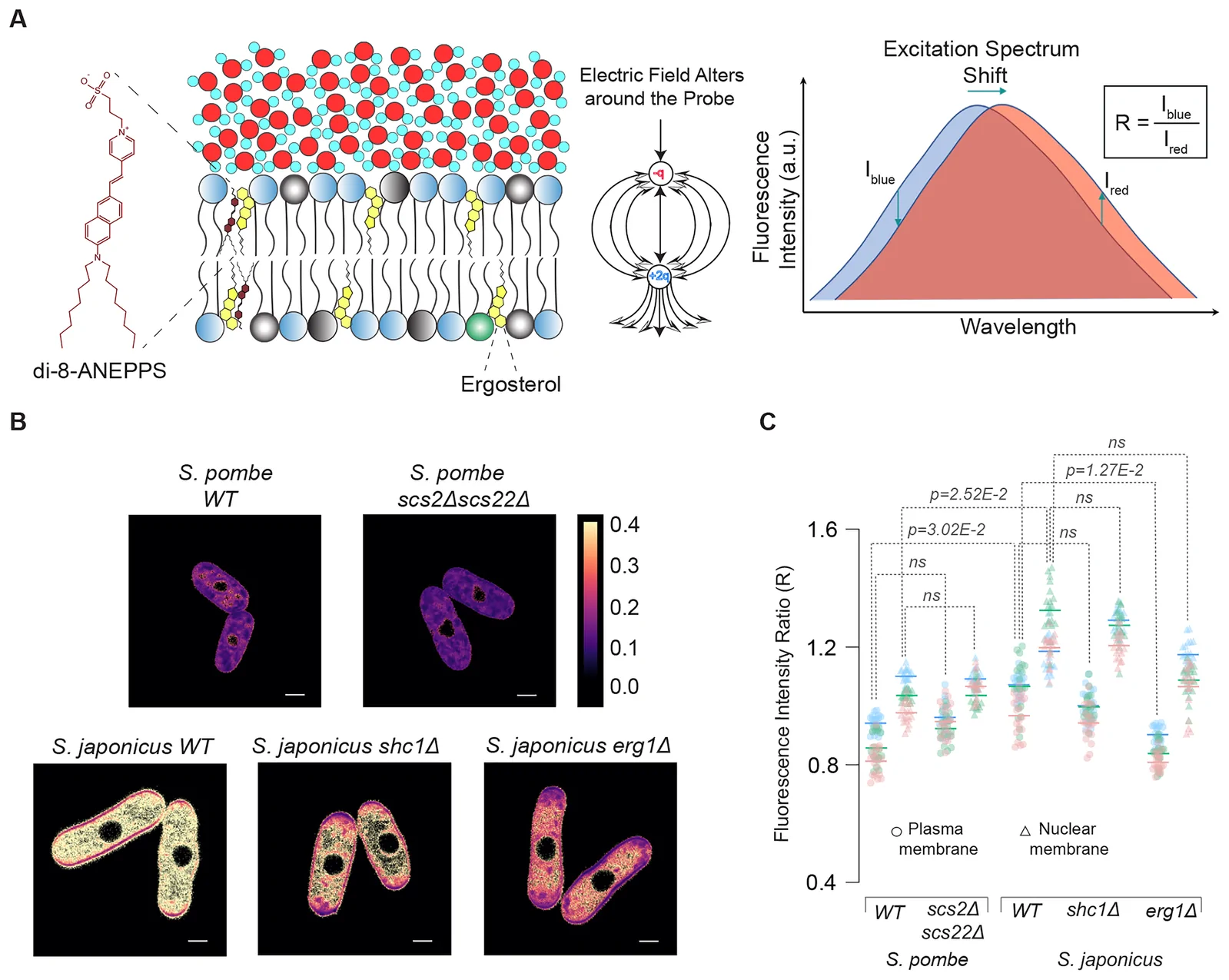
Ratiometric imaging reveals higher membrane dipole potential in *S. japonicus* cells as compared to *S. pombe*. (**A**) A schematic representation of the mechanism for estimating membrane dipole potential using the voltage-sensitive probe di-8-ANEPPS. Membrane bilayer composed of varied glycerophospholipid headgroups (light blue, grey and green) and sterol (yellow) incorporated with the probe. Dipole potential is generated due to the anisotropic arrangement of water (shown in proximity to the outer membrane leaflet for clarity) and lipid dipoles in the interfacial region. Charge transfer leads to a shift in the excitation spectrum of the probe, which is associated with changes in the local electric field strength. The final measurable parameter is the fluorescence intensity ratio (R), defined as the ratio of fluorescence intensities at the blue edge (I_blue_) and red edge (I_red_) of the excitation spectrum, and is proportional to the value of dipole potential. **(B**) Representative single-plane fluorescence intensity ratio (R) maps (color-coded in a scale of 0.0-0.4) in *S. pombe* and *S. japonicus* of indicated genotypes. R is defined as the ratio of fluorescence intensities at an excitation wavelength of 445 nm to that at 514 nm (emission band pass at 650-710 nm in both cases). Ratiometric images were generated using a custom Fiji macro and are shown as representative visual maps. Quantitative R values shown in (C) were calculated from the corresponding raw fluorescence images acquired at the two excitation wavelengths and not from the color-coded ratiometric maps. (**C**) A plot representing the individual values of fluorescence intensity ratios (R) calculated at the plasma membrane and at the NE/ER in cells of specified genotypes. Representative regions of interest (ROIs) are shown in Supplemental Fig. S2. The same ROI was applied to the corresponding images acquired at 445 and 514 nm, background fluorescence was subtracted separately in each channel, and R was calculated from the mean fluorescence intensities within the ROI. Data points represent measurements from individual cells from three independent biological replicates, with horizontal lines indicating the mean of each biological replicate. *p* values were obtained by two-tailed unpaired parametric *t*-tests, using the means of the three independent biological replicates (n = 3).

The dielectric environment in the interfacial region impacts on membrane dipole potential^41–43^. Membrane dipole potential is a positive electrostatic potential within the membrane, which originates due to the non-random arrangement of interfacial water and lipid dipoles^20,44–46^. Dipole potential can be measured by dual wavelength ratiometric imaging using voltage-sensitive dyes, such as di-8-ANEPPS^47–49^. The probe exhibits a shift in its excitation spectrum in response to changes in the surrounding electric field. A ratio of fluorescence intensities at the blue and red edge of the spectrum (R) thus represents the probe’s response to the electric field, indicating dipole potential (Fig. 2A).

We performed ratiometric fluorescence imaging of di-8-ANEPPS-labelled *S. pombe* and *S. japonicus* cells using confocal microscopy. In contrast to our FLIM measurements (Fig. 1), the fluorescence intensity ratio (R) at the plasma membrane of *S. pombe scs2Δscs22Δ* cells was not significantly different from that of the wild type (Fig. 2B, C). Interestingly, despite the FLIM measurements indicating comparable dielectric environments in *S. japonicus* and *S. pombe* membranes, the measurements of dipole potential indicated higher values in *S. japonicus* (Fig. 2B, C, see raw fluorescence images in Supplemental Fig. S2). Of note, the lack of ergosterol (*erg1Δ*) resulted in a significant reduction of dipole potential at the plasma membrane. In the absence of hopanoids (*shc1Δ*) the dipole potential measurements also trended lower but did not reach statistical significance.

### Ergosterol and saturated asymmetrical lipids support dipole potential

**Figure 3:**
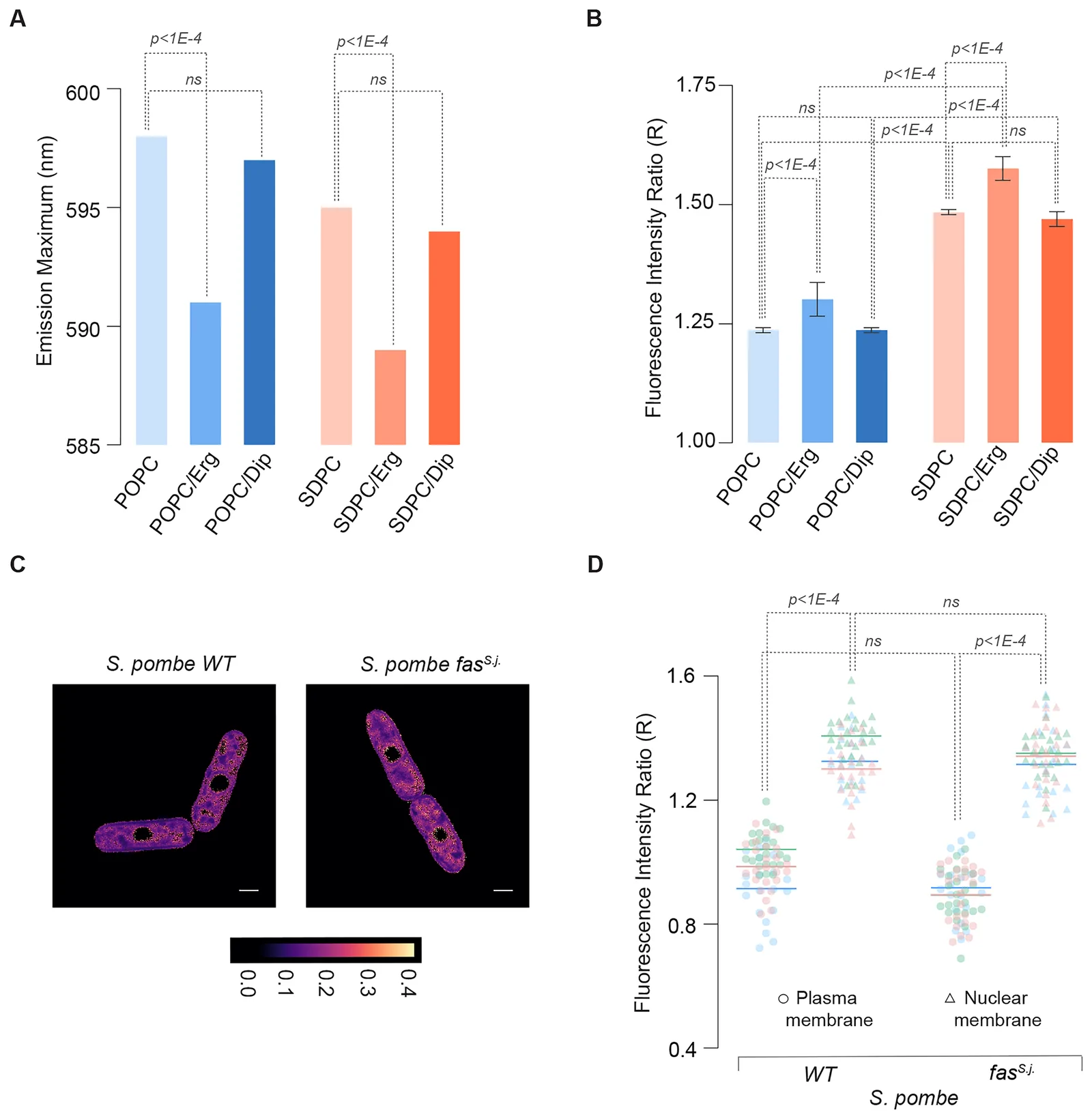
Ergosterol and saturated asymmetrical glycerophospholipids drive high membrane dipole potential. (**A, B**) Representative emission maximum (A) and fluorescence intensity ratio (R) values (B) of di-8-ANEPPS from spectroscopic measurements in single and two-component LUVs made with POPC or SDPC with either ergosterol (Erg) or diplopterol (Dip). Values represent average ± S.D. (n = 3 biological repeats). *p* values were obtained by two-tailed unpaired parametric *t*-tests. (**C**) Representative single-plane fluorescence intensity ratio (R) maps (color-coded in a scale of 0.0-0.4) in *S. pombe wild type* and *fas*^*S.j*.^ cells. (**D**) A plot representing the individual values of fluorescence intensity ratios (R) calculated at the cell tip regions of the plasma membrane and at the ER/NE in cells of specified genotypes. Representative regions of interest (ROIs) are shown in Supplemental Fig. S3. R values were obtained as described in Fig. 2. *p* values were obtained by two-tailed unpaired parametric *t*-tests, using the means of the three independent biological replicates (n = 3).

Given the complexity of biological membranes, we turned to artificial membranes in order to analyse the influence of glycerophospholipid architecture and fungal triterpenoids on membrane dipole potential. Using spectroscopy, we determined R values for di-8-ANEPPS-labelled single and two-component large unilamellar vesicles (LUVs) composed of POPC (C16:0/C18:1; monounsaturated symmetrical lipid) or SDPC (C18:0/C10:0; asymmetrical saturated lipid) in combination with ergosterol or diplopterol. We detected a blue shift in the probe’s emission maximum in two component LUVs of both POPC and SDPC in the presence of ergosterol but not diplopterol (Fig. 3A). This is indicative of a more hydrophobic ordered environment in membranes containing ergosterol and aligns with our earlier GP measurements using C-laurdan in two-component GUVs^14^ and in vivo dipole potential measurements (Fig. 2). Strikingly, SDPC exhibited a higher value of R relative to POPC in single as well as two component liposomes with ergosterol or diplopterol (Fig. 3B). Additionally, both POPC/ergosterol and SDPC/ergosterol liposomes showed increased values of fluorescence intensity ratio (R) relative to their counterpart two-component liposomes containing diplopterol. Although fluorescence-based imaging and cryo-EM measurements indicated that SDPC forms thin, disordered membranes^14^, the high dipole potential values suggest that SDPC membranes are relatively ordered in the interfacial region as compared to POPC-containing membranes. To disentangle the contribution of glycerophospholipid FA asymmetry vs FA unsaturation, we made use of *S. pombe* cells where both *fas2* and *fas1* genes encoding the α- and β-subunits of the cytosolic fatty acid synthase (FAS), were replaced with their *S. japonicus* orthologues (*fas*^*S.j*.^). The *fas*^*S.j*^ *S. pombe* mutant cells exhibit increased synthesis of the medium chain C10:0 FA and a concomitant increase in asymmetrical glycerophospholipids^12^. Unlike in *S. japonicus*, where most asymmetrical glycerophospholipids are saturated (C18:0-C10:0 and C16:0-C10:0), the asymmetrical glycerophospholipids in *fas*^*S.j*^ *S. pombe* are mono-unsaturated (C18:1-C10:0)^12^. Interestingly, dipole potential in *fas*^*S.j*^ *S. pombe* mutants was comparable to the wild type, suggesting that it is the glycerophospholipid FA saturation status rather than FA asymmetry that contributes to membrane dipole potential (Fig. 3C, D, see raw fluorescence images in Supplemental Fig. S3).

Taken together, our results indicate that in *S. japonicus*, the saturated asymmetrical glycerophospholipids together with the “native” triterpenoid ergosterol support high membrane dipole potential. The “foreign” triterpenoid diplopterol may have a relatively minor contribution to this parameter, at least in normoxia.

## Discussion

Membrane order, the dielectric environment and dipole potential are interrelated. High membrane order deep in the bilayer implies that dipole-manifesting groups are well aligned, leading to higher dipole potential. In disordered membranes, dipolar molecules would orient randomly resulting in lower dipole potential. Within the interfacial region, the relationship is not as straightforward. On the one hand, higher order reduces water penetration, yet water molecules may be ordered, yielding higher dipole potential. On the other hand, lower interfacial order allowing more water penetration may alter the orientation of dipolar molecules and the di-electric field, also increasing membrane dipole potential^50,51^. Although related, membrane physicochemical properties can vary independently based on local electrostatic effects and differences in lipid composition. Interestingly, previous di-4-ANEPPDHQ GP estimates produced higher values for *S. japonicus* membranes as compared to *S. pombe*^12,52^, whereas our FLIM measurements revealed comparable fluorescence lifetimes. Whereas GP is derived from changes in the steady-state emission spectrum of the probe, the fluorescence lifetime reflects its excited-state relaxation dynamics. Both are sensitive to the local membrane environment, where the relationship between lipid packing, hydration and polarity can be complex^21,31,53^. The two approaches therefore provide complementary readouts of membrane physicochemical properties.

Both ergosterol and diplopterol contribute to the maintenance of membrane polarity within the plasma membrane of *S. japonicus* (Fig. 1). Interestingly, we observed a reduction in di-4-ANEPPDHQ fluorescence lifetime at the NE/ER of *S. japonicus erg1Δ* cells relative to the wild type, but not in *shc1Δ* cells. Although sterols are enriched at the plasma membrane, they are synthesized in the ER – in fact, it was recently shown that the budding yeast ER membranes contain around 10% ergosterol^54^. Sterol concentrations of ~10% are often sufficient to support liquid-liquid phase separation in model membranes^55,56^, suggesting that even relatively modest ergosterol levels may contribute to the physicochemical organization of the NE/ER. Our results indicate that GP and fluorescence lifetime values need not to co-vary in complex cellular membranes.

Despite comparable di-4-ANEPPDHQ fluorescence lifetimes, the dipole potential is higher in *S. japonicus* membranes as compared to *S. pombe*, with ergosterol but not diplopterol being a major contributor to this parameter both in vivo and in model membranes (Fig. 2 and 3). Unlike sterols, diplopterol is less efficient at ordering the glycerophospholipids containing FA chains mono-unsaturated at position Δ-9^57^, which are the most prevalent unsaturated species in this organism^12,58^. In line with that, the aerobic physiology of *S. japonicus* relies largely on sterol production as compared to hopanoid biosynthesis, with *erg1Δ* mutant cells lacking sterols exhibiting a more pronounced decrease in cellular fitness vs *shc1Δ* cells^14^. However, in anaerobically growing *S. japonicus*, diplopterol fully substitutes for ergosterol. The shift towards a more saturated lipidome and increased abundance of asymmetric glycerophospholipids in the absence of oxygen^14^ may enhance the capacity of diplopterol to support membrane organization.

Of note, we observed a higher dipole potential in the NE/ER as compared to the plasma membrane in both fission yeast species. This observation is surprising because the ER membrane in mammalian cells showed lower membrane dipole potential relative to the plasma membrane^59^. In addition, such a higher dipole potential does not correlate with our GP^14^ and FLIM measurements (Fig. 1), which showed higher disorder and increased polarity in the NE/ER membranes. The enrichment in diacylglycerol and phosphatidic acid^32,60–62^, higher protein density and/or asymmetric lipid distribution in the NE/ER membranes may potentially contribute to local dipolar fields and increase the dipole potential. Importantly, our results using di-4-ANEPPDHQ and di-8-ANEPPS dyes imply that the relationship between closely related membrane biophysical properties is complex, and that measurements using different approaches should be interpreted with care^63^.

*S. japonicus* is a unique model eukaryote that has integrated hopanoids in its cellular metabolism and physiology. Together with its sister species *S. pombe*, it constitutes a powerful composite model system for probing the relationship between membrane lipid composition, physicochemical properties and the evolution of membrane proteins and membrane-centred biological processes. Indeed, we have previously shown that a subset of single-pass transmembrane proteins exhibit shortened transmembrane helices in *S. japonicus* as compared to other fission yeasts^12^. Higher membrane dipole potential in *S. japonicus* suggests that there could be additional molecular adaptations within the interfacial regions of transmembrane domains with potential implications for protein function, pointing to an interesting direction for future studies.

## Supplementary Material

Supporting Material

## Data Availability

Data generated during the current study are available from the corresponding author upon reasonable request.

## Why it Matters?

Our results lay important groundwork for further investigations of the relationship between the species-specific cellular lipid composition, membrane physicochemical properties and the evolution of membrane-associated proteins and membrane-centred biological processes.
