# Supplementary material for: Cellular membranes of the related fission yeast species differ in their dipole potential
Source: Biophys Rep (N Y). 2026 Aug 26;6(4):100283. doi: 10.1016/j.bpr.2026.100283 (PMC7619421; doi:10.1016/j.bpr.2026.100283)
Supplement: Supplementary file 1 [file mmc1.pdf]

Biophysical Reports, Volume *6*

Supplemental information

Cellular membranes of the related fission yeast species differ in their dipole potential

Bhagyashree Dasari Rao and Snezhana Oliferenko

Supporting Material

Cellular membranes of the related fission yeast species differ in their dipole potential

Bhagyashree Dasari Rao[1,2,3] and Snezhana Oliferenko[1,2,3]

[1]Randall Centre for Cell and Molecular Biophysics, School of Basic and Medical Biosciences, King's College London, London, SE1 1UL, UK
[2]The Francis Crick Institute, 1 Midland Road, London, NW1 1AT, UK

[3]Address correspondence to:
bhagyashree.rao@kcl.ac.uk or snezhka.oliferenko@crick.ac.uk

Supplemental Figures

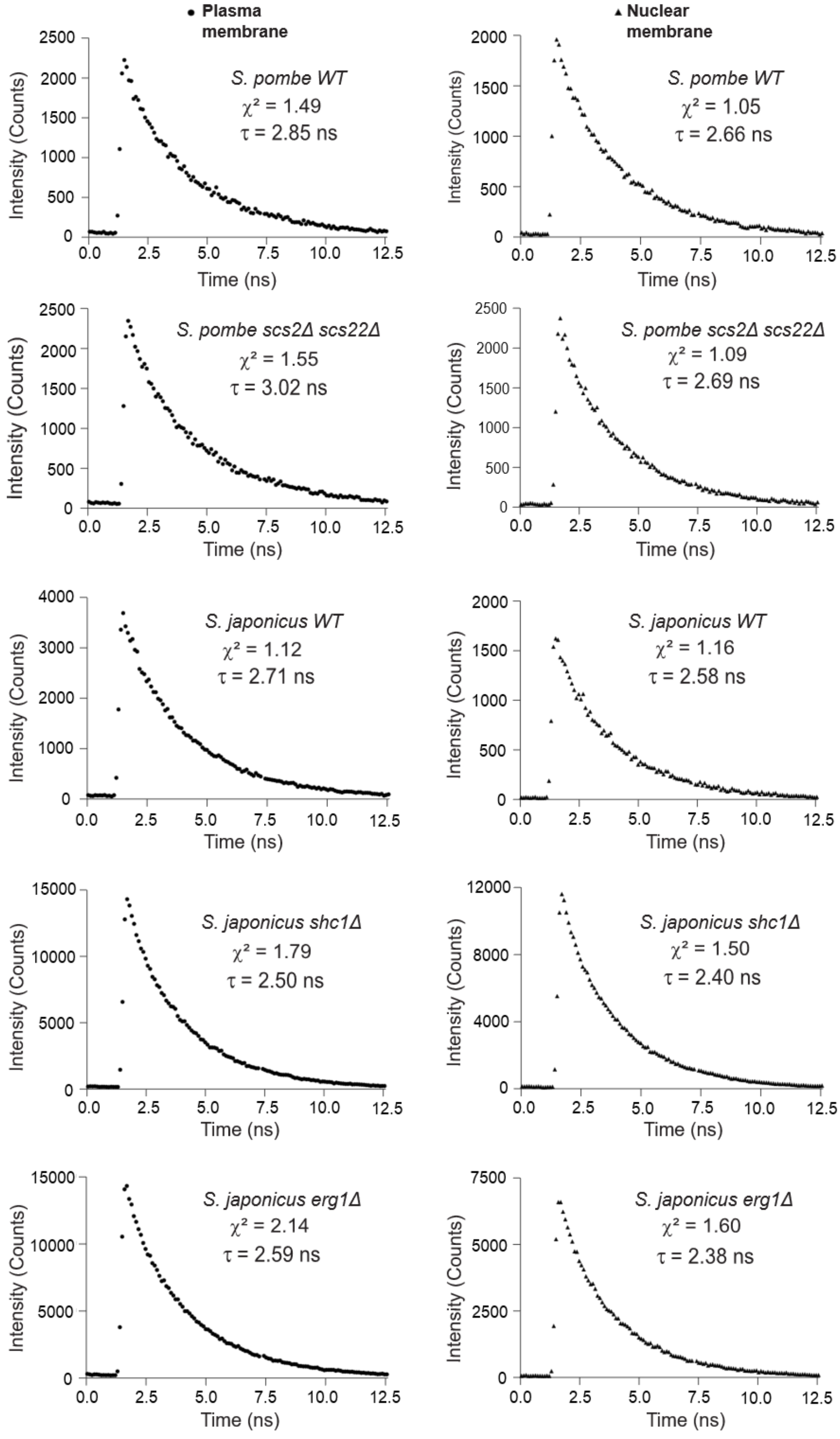

Figure S1. Representative fluorescence decay profiles for di-4-ANEPPDHQ FLIM measurements. Representative fluorescence decay profiles (intensity counts versus time) from the plasma membrane (circles) and nuclear membrane (triangles) ROIs corresponding to those indicated in Fig. 1A are shown for the indicated genotypes.

The fluorescence lifetime (τ) and χ^2 values shown for each ROI were obtained from the corresponding monoexponential fits performed in LAS X.

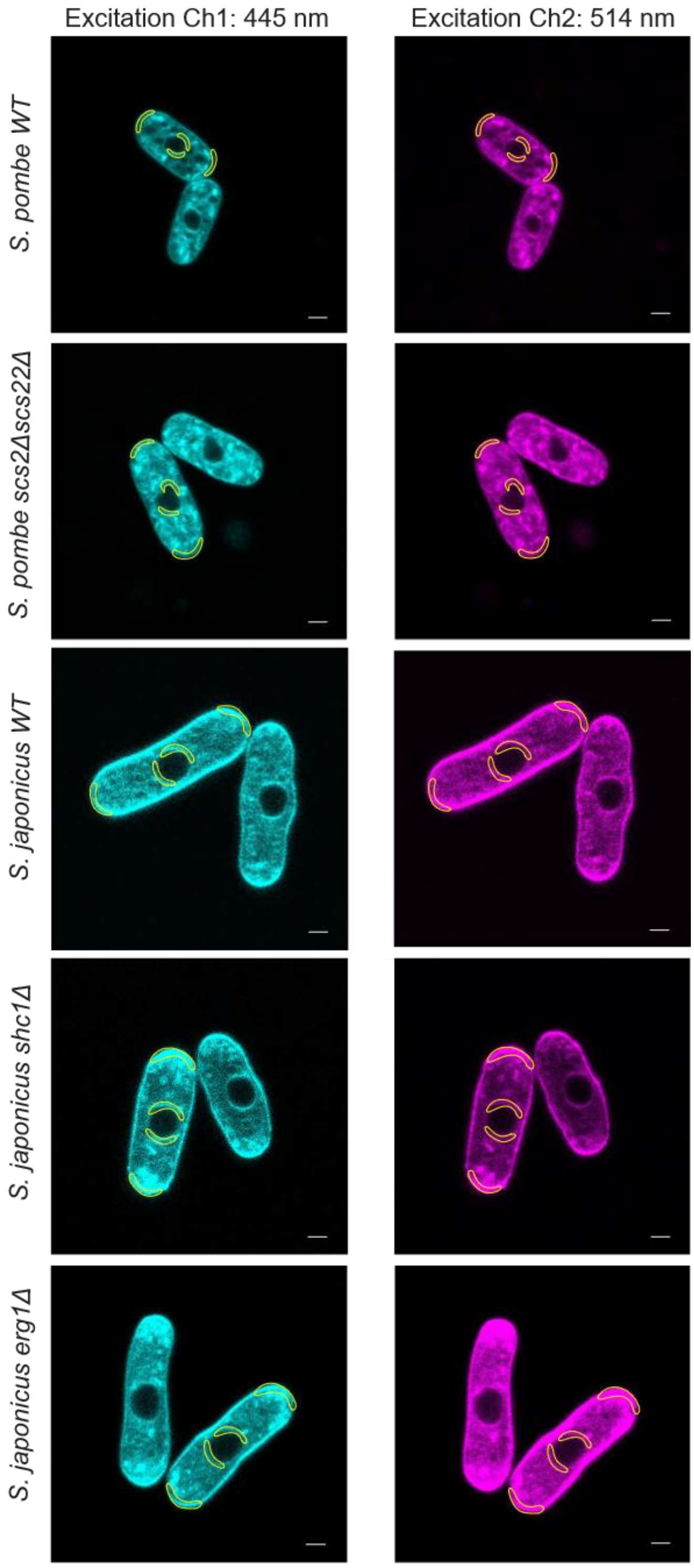

Figure S2. Confocal microscopic images of live fission yeast cells labelled with di-8-ANEPPS. Representative pseudocolor micrographs of live fission yeast cells at the two excitation wavelengths: λex = 445 nm (left panel), and λex = 514 nm (right panel). Regions of interest (ROIs) at the plasma membrane and the NE/ER membrane are highlighted. Scale bars represent 5 µm.

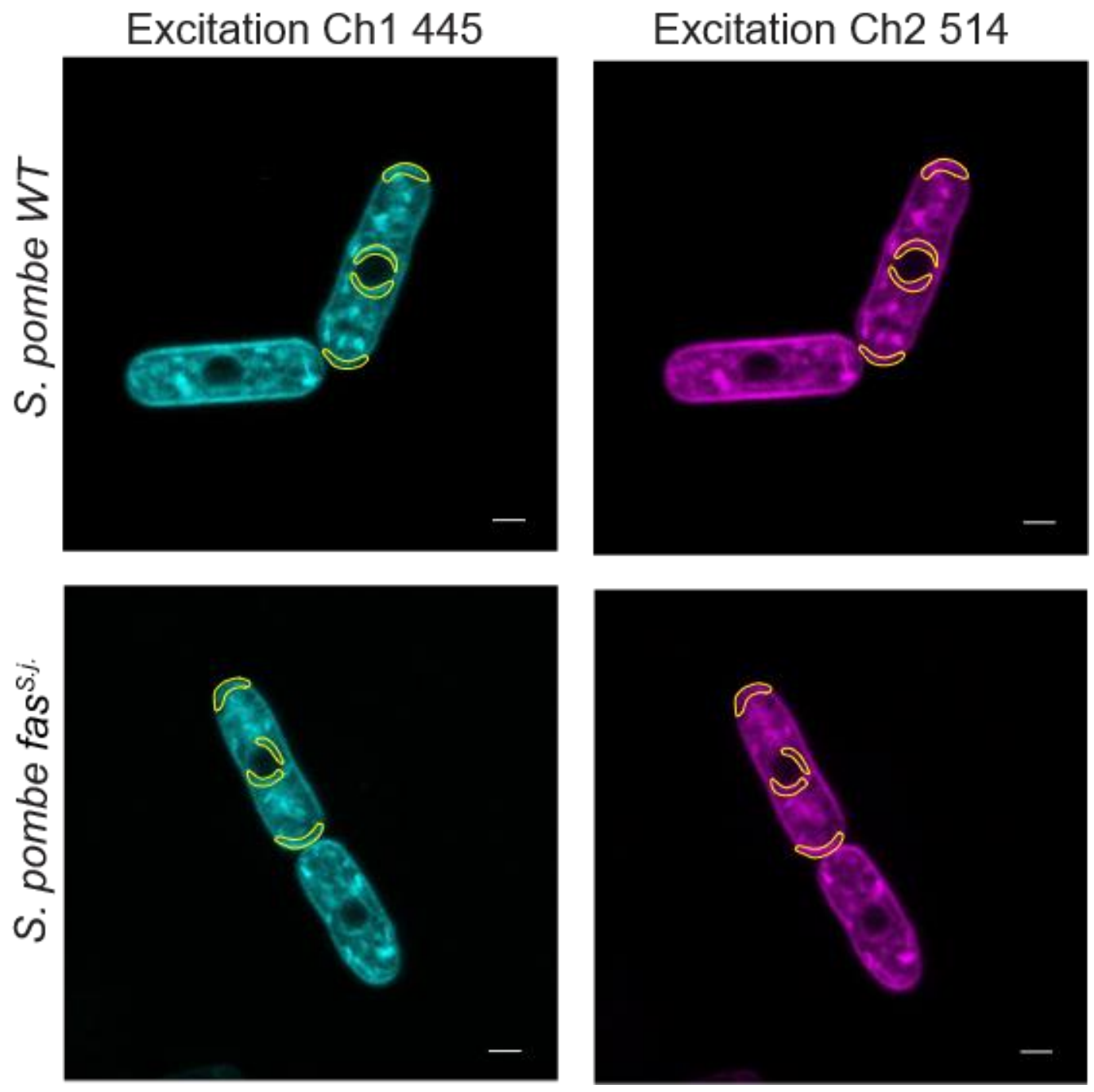

Figure S3. Confocal microscopic images of cells labelled with di-8-ANEPPS. Representative pseudocolor micrographs of *S. pombe* and *fas*$^{S.j}$ *S. pombe* mutant cells at the two excitation wavelengths: λex = 445 nm (left panel), and λex = 514 nm (right panel). ROIs at the plasma membrane and the NE/ER membrane are highlighted. Scale bars represent 5 µm.

Table S1. Fission yeast strains used in this study

**Strain**	**Genotype**	**Species**
SO8087	972 h- MBY99	*S. pombe*
SO6830	*scs22Δ::ura4+ scs2Δ::ura4+ h-*	*S. pombe*
SOJ2871	NIG2028<br>h- *matsj-P2028* (h- heterothallic isolate from NIG2021)	*S. japonicus*
SOJ5465	*shc1::kanMX6 ura4*+ h?	*S. japonicus*
SOJ5534	*erg1::natMX6* h?	*S. japonicus*
SO9285	MBY99 h+ prototroph	*S. pombe*
SO9277	*FASI S.j.:kanR + FASII S.j:hygR* (MBY99) h+	*S. pombe*
